# Molecular Vibration-Sensing Component in Human Olfaction

**DOI:** 10.1371/journal.pone.0055780

**Published:** 2013-01-25

**Authors:** Simon Gane, Dimitris Georganakis, Klio Maniati, Manolis Vamvakias, Nikitas Ragoussis, Efthimios M. C. Skoulakis, Luca Turin

**Affiliations:** 1 Royal National Throat, Nose and Ear Hospital, University College London, London, United Kingdom; 2 Vioryl S.A., Afidnes, Greece; 3 Neurobiology Division, Biomedical Sciences Research Centre “Alexander Fleming”, Vari, Greece; University of Arizona, United States of America

## Abstract

Whether olfaction recognizes odorants by their shape, their molecular vibrations, or both remains an open and controversial question. A convenient way to address it is to test for odor character differences between deuterated and undeuterated odorant isotopomers, since these have identical ground-state conformations but different vibrational modes. In a previous paper (Franco et al. (2011) Proc Natl Acad Sci USA 108:9, 3797-802) we showed that fruit flies can recognize the presence of deuterium in odorants by a vibrational mechanism. Here we address the question of whether humans too can distinguish deuterated and undeuterated odorants. A previous report (Keller and Vosshall (2004) Nat Neurosci 7:4, 337-8) indicated that naive subjects are incapable of distinguishing acetophenone and d-8 acetophenone. Here we confirm and extend those results to trained subjects and gas-chromatography [GC]-pure odorants. However, we also show that subjects easily distinguish deuterated and undeuterated musk odorants purified to GC-pure standard. These results are consistent with a vibrational component in human olfaction.

## Introduction

The human sense of smell uses the input from several hundred receptors to discriminate between tens of thousands of odorants. Although human olfactory receptors are members of the G-protein coupled receptor superfamily, the exact mechanism by which an odorant activates a receptor is still unclear. Specifically, we do not know whether olfactory receptors detect the shape of odorant molecules by a classical lock-and-key mechanism [1]–[4], their vibrations [5], [6] by a quantum mechanism [7]–[10], or a combination of both.

In principle, odorant isotopomers provide a possible test of shape vs. vibration mechanisms: replacing, for example, hydrogen with deuterium in an odorant leaves the ground-state conformation of the molecule unaltered while doubling atomic mass and so altering the frequency of all its vibrational modes to a greater or lesser extent [11]. To first order, deuteration should therefore have little or no effect on the smell character of an odorant recognized by shape, whereas deuterated isotopomers should smell different if a vibrational mechanism is involved.

The experimental evidence on this question to date is contradictory. Drosophila appears able to recognize the presence of deuterium in odorant isotopomers by a vibrational mechanism [12]. Partial deuteration of insect pheromones reduces electroantennogram response amplitudes [13]. Fish have been reported to be able to distinguish isotopomers of glycine by smell [14]. However, human trials using commercially available deuterated odorants [benzaldehyde and acetophenone] have yielded conflicting results, both positive [15] and negative [16]. Here, using GC-pure samples and a different experimental technique, we fully confirm Keller and Vosshall’s finding that humans, both naive and trained subjects, are unable to discriminate between acetophenone isotopomers.

However, since deuteration exerts the largest effect on the parts of the vibrational spectrum involving C-H motions, it seemed interesting to ask whether the effect of deuterium–if any–on smell character might be detectable in odorants containing more carbons, and therefore more CH groups. Musks are among the largest odorants and typically contain 15–18 carbons and 28 or more hydrogens [17], as compared to 8 carbons and 8 hydrogens for acetophenone. We now report that deuterated musks of diverse structures smell strikingly different from the parent compounds and similar to each other, even to naive subjects. The difference in smell character caused by deuteration persists when the most stringent criterion of purity, preparative gas-chromatography, is used.

## Methods

### Chemistry

#### Acetophenone

Acetophenone and d-8 acetophenone [99% D] were obtained from Aldrich and CDN Isotopes.

#### Musks

Deuterated musks are not commercially available, and total synthesis of deuterated musks from deuterated starting materials would be onerous in time and cost [18]. However, it is possible, using recently developed catalytic deuteration methods [19], [20], to partially deuterate the very pure musks that are commercially available. These methods do not give 100% deuteration yield, but involve mild reaction conditions [typically 160C and 5 atmospheres pressure] that create a minimum of unwanted side products. A musk suitable for deuteration should be available as a single molecule of high purity and must contain no double bonds, since the deuteration reaction is a hydrogenation. This narrows the choice among commercially available musks and led us to deuterate four molecules: three macrocyclic musks, cyclopentadecanone [Exaltone®], cyclopentadecanolide [Exaltolide®], 1,4-dioxa cyclohepta decane-5,17-dione [Astrotone®]; and, since aromatic rings are unaffected by catalytic deuteration, one polycyclic musk, 1-(3,5,5,6,8,8 -hexamethyl-6,7-dihydronaphthalen -2-yl) ethanone [Tonalid®].

#### Procedure for the H-D exchange reaction of musks

**Figure 1:**
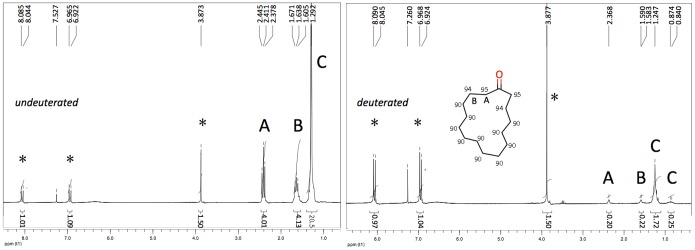
NMR spectra of deuterated cyclopentadecanone in CDCl_3_. Left: H- cyclopentadecanone. The peaks indicated by asterisks are those of the internal standard, p-anisic acid. Protons can clearly be resolved into 4xA, 4xB and the remainder [20 protons]. Right: Deuterated cyclopentadecanone. The ^1^H signal is greatly reduced. From the ratio of the integrated ^1^H signal before and after deuteration we can calculate the yield to be 95% deuteration in the A position and 94% in the B position. The remaining protons are deuterated to 90%. No impurities are seen in the spectra.

**Figure 2:**
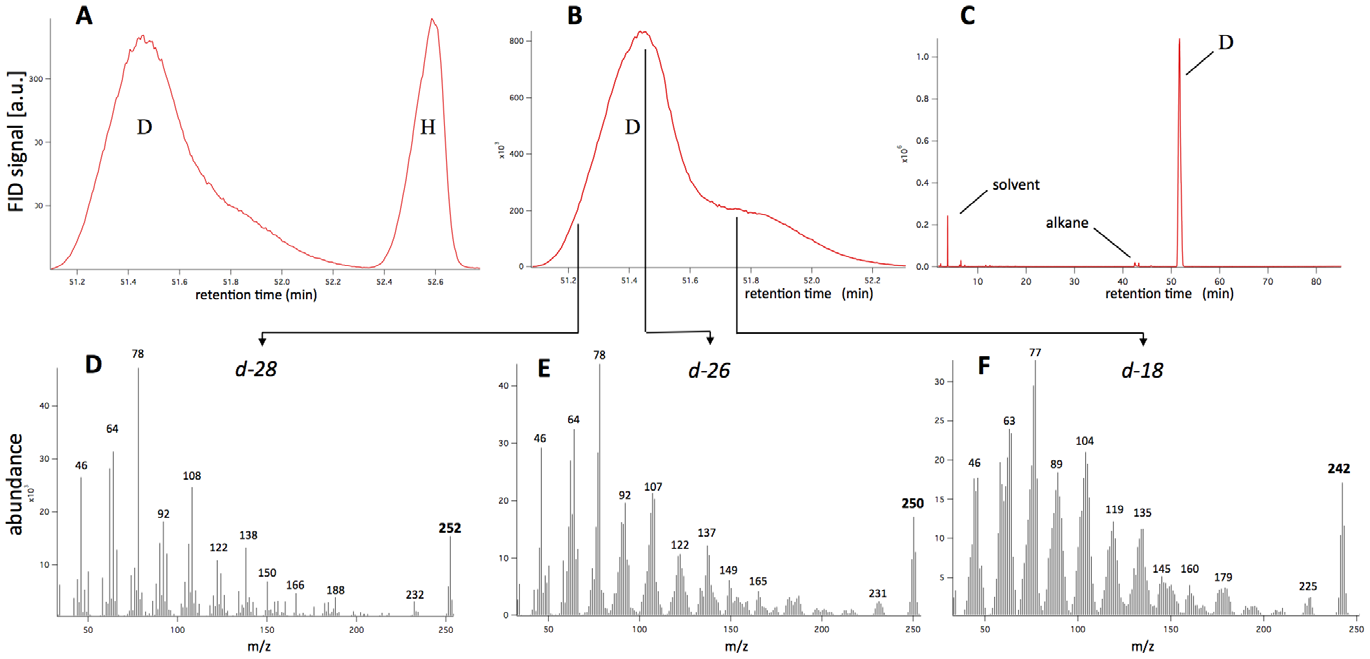
GC and MS spectra of deuterated cyclopentadecanone. A: GC trace of a deliberate, approximately equimolar mixture of deuterated [D] and undeuterated [H] cyclopentadecanone. Good chromatographic separation is obtained, with a difference in retention time of approximately 1.2 minutes. B: GC trace of a different sample of deuterated cyclopentadecanone, coupled to mass spectrometry. C: Full GC trace of a sample of deuterated cyclopentadecanone after silica gel purification. The solvent peaks at retention times <15 min amount to 5% of total, and the alkane peaks [identified by MS] at 42–43 minutes amount to less than 1%. Peak D is then repurified by preparative GC and used in the smelling tests. D, E and F: Mass spectra obtained by sampling B at 51.21, 51.42 and 51.76 minutes. The units of the abscissa are daltons/unit charge. Consistent with panel A where the deuterated cyclopentadecanone exits the column before the undeuterated exaltone, the perdeuterated species [d-28] exits the column first followed by less deuterated d-26 and d-18 fractions.

A mixture of cyclopentadecanone (2.8 mmol, 628 mg, Aldrich) diluted in 500 µl of cyclohexane, used as a co-solvent to dissolve the substrate according to [20], D_2_O (12ml, Aldrich) and 5% Rh/C (126 mg, 20 wt% substrate, Aldrich) was prepared. The reaction was carried out in a Q-Tube™ (QLabtech, Tamarack, CT) pressure vessel under a pressure of 50 psi. The reaction mixture was stirred at 160 C under an H_2_ atmosphere for 48 h. After the reaction was complete, the mixture was extracted with Et_2_O and passed through a membrane filter to remove the catalyst. The ethereal layer was washed with brine, dried over MgSO_4_ and concentrated in vacuo. The residue was purified by chromatography (hexane:ether = 9∶1) on silica gel to afford the deuterated product (370 mg). The deuterium content of the product was calculated by using ^1^H NMR spectra (Varian, 200 MHz) with *p*-anisic acid as an internal standard [Figure 1]. The amount of deuterium incorporated was also determined by Gas Chromatography-Mass Spectrometry (GC: HP 6890 Series; MS: Hewlett Packard-MSD 5973) [Figure 2].

#### Sham deuteration of musks

The same procedure as above was followed except that D_2_O was replaced by H_2_O.

#### Data for D-Exaltone

^1^H NMR (CDCl_3_, p-Anisic acid 0.5 equivalents as an internal standard) δ 2.37 (m,0.2H), 1.59 (m,0.22H), 1.30-1.20 (m,1.72H) 0.84–0.87 (m,0.25H).

#### Results of deuteration reaction

Each musk behaved differently during deuteration. Exaltone gave the highest yield and the fewest side products [a few percent, chiefly cyclopentadecane and cyclopentadecanol, the latter easily removed by silica gel chromatography]. Exaltone is also powerful and easily detected at 1 mg level on the smell port of a smelling gas chromatograph [GC]. Exaltolide had low deuteration yields, always less than 50% and rather variable from reaction to reaction. Astrotone had good deuteration yields but required temperatures >250C to reliably exit the capture port of the GC, which is close to its decomposition temperature. Tonalid was fully deuterated only in 9 positions and yielded many side products.

#### Odor evaluation of musks

After silica gel purification, aliquots of the deuterated musks were diluted in ethanol and their odor character assessed on smelling strips. The parent compounds have classic powerful musk odor characters, with secondary perfumer descriptors as follows: animalic [Exaltone], sweet [Exaltolide], oily [Astrotone] and sweet [Tonalid]. In all the deuterated musks, the musk character, though still present was much reduced, and a new character appeared, variously described by the trained evaluators [NR, DG, LT and Christina Koutsoudaki, Vioryl SA] as “burnt,” “roasted,” “toasted,” or “nutty.” Naive subjects most commonly described the additional common character as “burnt.” Sham-deuterated musks smelled the same as the starting material.

#### Partially deuterated musks

**Figure 3:**
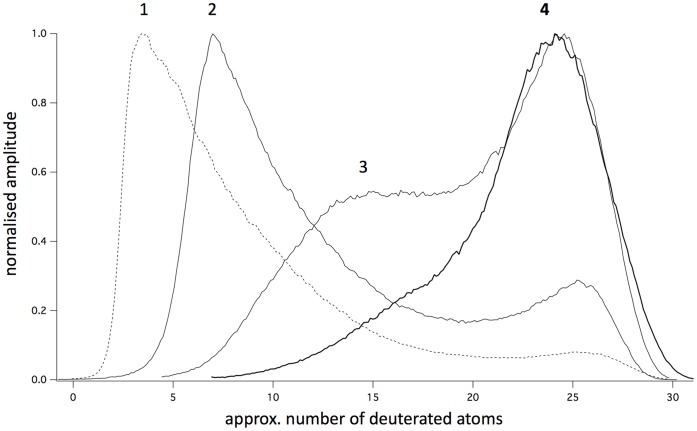
GC elution profiles of partially deuterated cyclopentadecanone samples. The GC traces have been normalised to a peak amplitude of 1 and are shown plotted against approximate deuteration number rather than retention time as in figure 2. The deuteration scale is taken from mass spectra taken at different times and differs slightly between one sample and the next because of slight differences in the GC elution times. The abscissa should therefore be taken as approximate, i.e +/−2 deuterated atoms. Trace 4 [bold] is taken from figure 2 A. The sample depicted in trace 1 had no discernible burnt odor character.

It seemed interesting to ask at what intermediate level of deuteration the burnt character made its appearance. To this end we prepared samples of cyclopentadecanone deuterated to differing extents, by stopping the reaction before complete deuteration was achieved. The normalized GC traces corresponding to each sample are shown in figure 3. The samples were purified and evaluated by trained observers as above. The deuterated character was undetectable only in the sample with the lowest percentage of deuteration [sample 1] and present in the others, suggesting that less than 50% deuteration [14 deuteriums] is insufficient to change odor character. The wide range of deuteration present in all samples prevents a more accurate estimate at this time.

### Further Purification

To establish unequivocally that a given odor character belongs to a particular molecule, ideally *GC-pure* samples must be smelled *double-blind*. GC-smelling is usually done by fitting a chromatograph with a smelling port separate from the detector port. As the peaks exit the column, they can be smelled from the smelling port. The advantage of GC smelling is essentially ideal purity if peaks are well separated. The disadvantage is the necessity to memorize a smell character, typically for several minutes, for comparison with that of a later GC peak. In practice, conventional GC-smelling is a skill suited only to A/B comparisons by experts.

An elegant solution to this problem was devised some years ago by Dr Christina Zelano (UC Berkeley). As in so-called preparative GC [21], the puff of odorant exiting the smelling port can be condensed in, for example, a polyethylene Eppendorf centrifuge tube of 1 ml capacity, which is brought up to the smelling port when the relevant GC peak begins and removed and sealed when it ends. Provided the odorant is powerful enough, the tube can be reopened one or more times later and the odorant trapped in it assessed for odor character in comparison with another odorant trapped in a second tube.

The sample preparation method was standard for all the tested odorants: in an HP 5830 gas chromatograph with a flame ionization detector, using helium as makeup gas with a 15 m column and splitter adaptor with an output port upon which a collecting tube could be placed. A 5 µl aliquot of the substance was injected into the inlet. Only odorants of 98% purity or higher were used, to reduce the likelihood of contaminants with equal transit times through the column. The sample peak was detected at the flame ionization detector [FID] and captured at the port with an approximately 1-second lag between detection at the FID and the substance being detectable by smell at the port. The capture tube was positioned on the output port as soon as the FID trace began to move, and removed and sealed when the FID trace fell back to baseline. The resolution and time were such that there were no coincident peaks within at least 5s of the sample peak.

#### Acetophenone

Standard capped Eppendorf low-density polyethylene tubes were packed with approximately 1 cm^3^ of sterile cotton wool. All contact was with clean latex clinical gloves and acetone cleaned instruments. One peak was captured into each tube, which was then capped and stored at −4°C until use.

#### Musks

Adjustments were necessary to adapt the method to musks. (1)- It was necessary to capture several–four were sufficient–GC peaks from different runs in the same capped Eppendorf LDPE tube for the odor intensity to be sufficiently strong to give a clear odor character. (2)- For better odor retention at room temperature and above, the tubes were coated internally with a very thin layer of petroleum jelly, which captured more odorant at the expense of lowering its vapor pressure. The petroleum jelly layer was formed by the addition of a solution of commercial Vaseline® in low boiling point petroleum ether. 1 ml was added to the tubes and shaken, the excess poured off and the petroleum ether allowed to evaporate at room temperature for 30 min. The tubes were then odorless. (3)- To offset the lower vapor pressure, the loaded tubes were set on a heated block a few minutes before smelling tests and kept at 50C during the tests to saturate the internal headspace with odorant. Of the deuterated musks, we found that cyclopentadecanone [Exaltone] was optimal, being highly deuterated, thermally stable and powerful.

### Human Test Protocol

#### Ethical approval

All human tests were carried out under the approval of the UCL Research Ethics Committee number ID 1585/001. All subjects signed a written informed consent as per UCL Research Ethics guidelines.

#### Subject preparation

Before undertaking any trials, each subject undertook a standard University of Pennsylvania Smell Identification Test [UPSIT] test and a Sino-Nasal Outcome Test [SNOT-22] questionnaire to exclude obvious nasal disease. Any with severe olfactory impairment on either test were excluded from further study. For musk trials, each subject was screened for musk anosmia by being presented with a non-deuterated musk sample and asked if they could smell anything. Failure to detect an odor in either of two presented samples excluded subjects from any further trials.

#### Blinding

To double blind the experimenter and volunteer, each Eppendorf was labelled with a UV-visible ink. Each sample was assigned a unique identifier number for that trial, marked on the cap of the Eppendorf tube. The number was not visible in normal light, but under a portable UV light source (Helix) the sample number could be identified. The samples were thus indistinguishable to both experimenter and volunteer until after the volunteer had smelled and handed them back to the experimenter. The number could then be recorded and the nature of the sample known.

## Results

### Acetophenone

All trials were performed with Gas Chromatography-pure [GC-pure] commercially available acetophenone and perdeuterated d8-acetophenone [See Methods]. Each trial set consisted of a total of 20 samples, 10 each of the deuterated and undeuterated compound, produced in alternating batches of 5 samples per odorant. The samples were mixed together in a cardboard box, which was thoroughly shaken before every sample was blindly selected.

The sample was smelled, identified and replaced before the box was shaken again. This requirement of random selection *with replacement* places the chance of any pairwise comparison being the same or different at 50%.

For each trial pair the subject had merely to state whether the smell character was the same or different, a same-different forced choice test. 5 subjects were recruited, 3 perfumers and 2 untrained. Multiple trials of 10 pairs per trial were undertaken as tolerated, some on different days with the same subject.

The results are presented in Table 1. Only one subject [SG] achieved a binomial p less than 0.01, and that only after 399 trials. The others ranged between 0.035 and 0.086. These results indicate that selection of the correct choice was by chance alone and are consistent with the conclusion that, under these experimental conditions, humans are unable to discriminate acetophenone from perdeuterated acetophenone.

**Table 1:** Results of human discrimination tests between GC-pure deuterated and undeuterated acetophenone.

Subject	TG	DR	TS	AD	SG
numbercorrect	39	44	172	154	217
numberof trials	80	80	350	320	399
%correct	48.75%	55.00%	49.14%	48.13%	54.39%
binomial p	0.086	0.059	0.04	0.035	0.008

The success rate in all cases was close to 50%, indicating that the subjects did not do better than chance.

### Musks

All trials were performed with GC-pure catalytically deuterated [D fraction >90%] cyclopentadecanone [See Methods]. Each trial consisted of the assessment of 4 pairs of odorants, one deuterated and one sham-deuterated. The subjects were presented with a deuterated sample and their attention was drawn to the “burnt, nutty, roasted” character of the deuterated compound. Several other sample pairs were presented until the subjects were sure they could tell the difference between the two sample types.

The Eppendorf tubes were heated in a solid heating block to 50C. The samples were arranged in rows according to their type. The experimenter randomized the order of the tubes within the rows by means of two flips of a coin (first flip: first or second two positions, second flip: first or second spot within those). The rows were then mixed randomly by a further coin flip per d/H pair (heads: swap positions, tails leave in situ).

Subjects were first given a training pair and told which was deuterated and which sham-deuterated. The experimenter then left to watch the experiment through a window. Subjects were then presented with the unlabeled, position-randomized pairs of deuterated and sham-deuterated GC-pure samples and asked to say which was which.

The subject, wearing nitrile gloves to avoid contamination, smelled first one and then the other sample. Multiple sniffs at each sample were allowed. The subject was asked to identify the deuterated sample and to place it to one side. After four trials the tubes were placed under the UV light source and identified. The subject was not informed of the outcome. To avoid habituation, the subject then rested for 15 minutes before attempting the next trial.

The results are shown in table 2. Eleven subjects were used. Two subjects tired before reaching the desired number of 12 trials. Two were able to go beyond to 13 and 17 trials respectively. The binomial p values range between 0.109 [6/8 correct] to 7.62×10^−6^ [17/17 trials]. These are independent trials, and an aggregate probability for all trials [119/132 correct] can be calculated: it is equal to 5.9×10^−23.^

**Table 2:** Results of human discrimination tests between GC-pure deuterated and sham-deuterated cyclopentadecanone.

Subject	MG	LT	KF	KM	NH	CC	AM	CS	JB	VC	AD
numbercorrect	9	12	6	17	12	9	12	11	12	9	10
numberof trials	10	12	8	17	12	12	13	12	12	12	12
binomial p	0.0097	0.0002	0.1094	7.00E-06	0.0002	0.0537	0.0016	0.0029	0.0002	0.0537	0.0161

Tests were halted when the subject reported fatigue. The binomial probability of the successes having arisen by chance is indicated at the bottom of each column. See text for details of method.

## Discussion

Our results indicate that subjects cannot differentiate GC-pure undeuterated acetophenone from deuterated acetophenone. This is inconsistent with a previous report by one of us [LT] that the two GC-pure odorants smell different [7] and consistent with a later report that they do not [16]. It is likely that reports of odor differences between acetophenone isotopomers [15] that were not GC pure were due to residual impurities. Remarkably, however, naive subjects can differentiate between undeuterated [sham-deuterated] cyclopentadecanone and deuterated cyclopentadecanone with a high degree of accuracy. In addition, all the subjects who could smell the musks reported the same additional character being present in GC-pure deuterated cyclopentadecanone that trained evaluators found in the other three deuterated musks: nutty, roasted, burnt.

The main confounding factor in comparisons between odorants is usually impurities, which, even at low levels, can significantly change odor character. Our method reduces this problem to the feasible minimum. First, the starting materials are very pure already, since commercial fragrance materials avoid impurities for precisely the same reason. Second, the deuteration method is mild and, when applied to macrocyclic musks, appears to introduce no side-products to the pure starting material aside from partial reduction of the keto group to an alcohol. Third, sham-deuteration causes no change in odor character, indicating that no odorant impurities are being introduced by the heating of the reagents and apparatus. Fourth, the GC purification method singles out the main peak and would only let through impurities fortuitously endowed with the same mobility in the GC column, for which there is no evidence in NMR spectra. Finally, the commonality in odor character among the four musks would require the same impurity to arise as a reaction product of an acetylated tetralin, a lactone, a dilactone and a ketone, which is unlikely. We therefore feel confident that an impurity is not responsible for the odor of deuterated musks.

Another possibility might be that the odor difference, while not due to impurities, is caused by some subtle physicochemical difference common to deuterated musks, which is interpreted by the olfactory system as a burnt character. There is no question that small differences do exist between deuterium and hydrogen compounds. For example, dispersion forces are a bit weaker in deuterated molecules, as evidenced by the fact that deuterated cyclopentadecanone, though higher in molecular weight, exits the GC column approximately 1.2 minutes *before* its normal counterparts [see figure 2A]. This said, the difference in retention times among different non-deuterated musks is even larger. For example, cyclotetradecanone and cyclohexadecanone, two other macrocyclic ketones, would respectively exit the GC column used in these experiments approximately 3 minutes before and after cyclopentadecanone. Such relatively larger differences in dispersion forces among these musks might be expected to manifest themselves in a small change in affinity of the musk for the receptor and therefore alter their odor character. Yet both smell musky and utterly lack the burnt character associated with deuterated musks. [22], [23]. Therefore it is hard to see how such differences could be responsible for the change in odor character of the deuterated cyclopentadecanone, which is perceptible over a large range of concentrations, from pure to dilute GC puff. Finally and more generally, it seems very unlikely that a small change in any hypothetical physicochemical property would add the same–burnt, roasted–smell character in several different molecules with differing shapes and solubility properties.

By contrast, this commonality of odor character is precisely what is expected of a vibrational mechanism. Regardless of molecular shape and connectivity, the bands of C-H stretch, wag and scissor modes will shift predictably and collectively in frequency between hydrogen and deuterium isotopomers. In similar fashion, vibration theory can successfully account for the commonality of the odor of molecules of different structures that contain the same functional groups [sulfuraceous, nitric, ethereal, etc.], a phenomenon both familiar and otherwise unexplained by shape theory [24]. Furthermore, vibrational spectra appear to be a better predictor of odor character than shape descriptors [25]–[27]. However, the question of which vibrational band might be responsible for the “deuterated” character is difficult to answer at present. Our studies on Drosophila [28] have shown that fruit flies can generalize avoidance from a nitrile to a deuterated odorant of unrelated structure and vice versa. This is difficult to explain except by assuming that Drosophila perceives the ≈2150 cm-1 vibrational band, common to both, as having a distinctive odor. It is conceivable that a similar effect is seen in our experiments and that the harsh character of deuterated samples is due to a small amount of nitrilic character, usually described as harsh, oily and metallic [29]. Alternatively, it could be due to spectral changes in the fingerprint region. Until generalization experiments are performed with human subjects, the assignment of the odor character of deuterated musks to a particular spectral band remains open.

Why does deuteration substantially alter the odor character of musks but not that of acetophenone? If, as has been proposed, an inelastic electron scattering mechanism is at work, it will be very sensitive to partial charges [8], [9], [30]. The C-H bond is weakly polar. A bond of low polarity may be difficult to detect by smell. This is consistent with most alkanes having weak smells, homonuclear diatomic gases being odorless, B-H bonds on carboranes being undetectable [8], and cyanohydrins having no nitrilic character [and small or absent C≡N stretch bands in the infrared [31] ]. Therefore it may be that there must be many C-H bonds before they are detectable by smell. In contrast to acetophenone which contains only 8 hydrogens, cyclopentadecanone has 28. This results in more than 3 times the number of vibrational modes involving hydrogens than in acetophenone, and this is likely essential for detecting the difference between isotopomers. The relationship between carbon number and deuteration effect on odor character will be studied more closely in further work.

One enduring puzzle of structure-odor relations is the relationship between musk odor [typically very similar from musk to musk] and molecular structure [very diverse] [32]–[36]. To this puzzle we tentatively offer a novel solution. Musks are at the upper end of the molecular weight scale for odorants. An empirical rule of fragrance chemistry stipulates that molecules made up of more than 18 carbons tend to be odorless. This is not due to volatility, since heating an odorless 19-carbon compound will not make it smell. Instead, it appears to be a size limit of the receptors themselves. At the upper end of the odorant molecular weight range, many subjects become anosmic [37]–[38]. One of us [EMCS] is completely anosmic to all the musks used in this paper, as was one of the prospective subjects tested with Exaltone. This, as well as *in* *vitro* studies of receptor selectivity [39], suggests that a small number of receptors, possibly just one, are involved in sensing musk odor.

If olfaction has a vibrational component, then receptors will likely be tuned to one part of the vibrational range. Which part of the vibrational spectrum would a musk receptor be sensitive to? It has long been known [40] that nitration of benzene rings substituted with bulky t-butyl groups yields excellent “nitro” musks, the historical precursors of the musks used in this article. Remarkably, the very intense symmetric and antisymmetric stretch vibrations of nitro groups at ≈1380 and ≈1550 cm-1 rather closely match prominent scissor and wag modes of C-H moieties. We suggest therefore that a musk odor is achieved when three conditions are simultaneously fulfilled: First, the molecule is so large that only one or a very few receptors are activated. Second, one or more of these receptors detects vibrations in the 1380–1550 cm-1 range. Third, the molecule has intense bands in that region, caused either by a few nitro groups or, equivalently, many CH_2_ groups. A properly quantitative account of musk odor will require better understanding of the shape selectivity of the receptors at the upper end of the molecular weight scale, and of the selection rules of a biological IETS spectrometer [8]–[10], [26] to calculate the intensity of vibrational modes. We propose that the reason for the close resemblance among the odor character of musks of widely different structures may therefore be entirely biological: to first order, only one receptor for a particular band of the spectrum is activated and musk odor character is thus essentially, perhaps uniquely, “monochromatic.”
