# Correction: Molecular Vibration-Sensing Component in Human Olfaction

**DOI:** 10.1371/annotation/2f278ed8-d5e7-440a-9e49-c8d1df20d1f1

**Published:** 2013-05-31

**Authors:** Simon Gane, Dimitris Georganakis, Klio Maniati, Manolis Vamvakias, Nikitas Ragoussis, Efthimios M. C. Skoulakis, Luca Turin

Upon a re-analysis of the data, there were errors in the p-values reported in Table 1 and Table 2. The revised p-values for Tables 1 and 2 are as follows:

Table 1 Individual p-values: TG: 0.910 ; DR: 0.434; TS: 0.79; AD: 0.539; SG: 0.089. Aggregated p-value of 0.512

Table 2 Individual p-values: MG: 0.021; LT: 0.0004; KF: 0.2891; KM: 1.54 10-5; NH: 0.0004; CC: 0.146; AM: 0.0034; CS: 0.0063; JB: 0.0004; VC: 0.146; AD: 0.0385. Aggregate probability : < 2.2 10-16.

The full, corrected Table 1 can be viewed here: 

**Figure:**
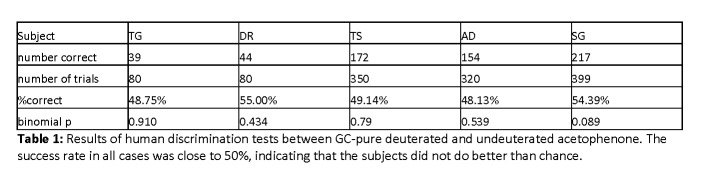


The full, corrected Table 2 can be viewed here: 

**Figure:**
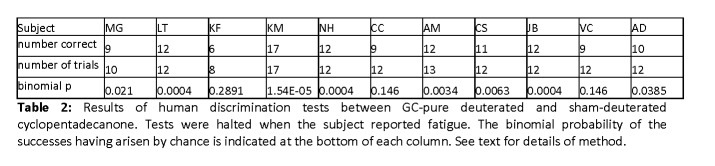


The results with acetophenone are indistinguishable from chance; those with cyclopentadecanone are highly statistically significant. Those were the main findings of the article and they remain unchanged by the modifications noted above.

In addition, the funding source in the acknowledgments section was incompletely specified. The full description of funding sources is as follows:

Funded by the Greek Ministry of Education and Research, General Secretariat for Research and Technology under the framework ESPA 2013-2017, program ARISTEIA 2303 to EMCS.

